# Structural and Functional Brain Abnormalities Associated With Exposure to Different Childhood Trauma Subtypes: A Systematic Review of Neuroimaging Findings

**DOI:** 10.3389/fpsyt.2018.00329

**Published:** 2018-08-03

**Authors:** Laura L. M. Cassiers, Bernard G. C. Sabbe, Lianne Schmaal, Dick J. Veltman, Brenda W. J. H. Penninx, Filip Van Den Eede

**Affiliations:** ^1^Collaborative Antwerp Psychiatric Research Institute, University of Antwerp, Antwerp, Belgium; ^2^University Department of Psychiatry, Campus Antwerp University Hospital, Antwerp, Belgium; ^3^University Department of Psychiatry, Campus Psychiatric Hospital Duffel, Antwerp, Belgium; ^4^Orygen, The National Centre of Excellence in Youth Mental Health, Parkville, VIC, Australia; ^5^Centre for Youth Mental Health, University of Melbourne, Melbourne, VIC, Australia; ^6^Department of Psychiatry, VU University Medical Center, Amsterdam, Netherlands; ^7^Amsterdam Neuroscience, VU University Medical Center, Amsterdam, Netherlands

**Keywords:** child abuse [MeSH], neuroimaging[MeSH], childhood trauma, trauma subtypes, neglect, sexual abuse, emotional maltreatment, physical abuse

## Abstract

**Background:** Childhood trauma subtypes sexual abuse, physical abuse, emotional maltreatment, and neglect may have differential effects on the brain that persist into adulthood. A systematic review of neuroimaging findings supporting these differential effects is as yet lacking.

**Objectives:** The present systematic review aims to summarize the findings of controlled neuroimaging trials regarding long-term differential effects of trauma subtypes on the human brain.

**Methods:** A systematic literature search was performed using the PubMed and PsycINFO databases from January 2017 up to and including January 2018. Additional papers were identified by a manual search in the reference lists of selected papers and of relevant review articles retrieved by the initial database search. Studies were then assessed for eligibility by the first author. Only original human studies directly comparing neuroimaging findings of exposed and unexposed individuals to well-defined emotional, physical or sexual childhood maltreatment while controlling for the effects of other subtypes were included. A visual summary of extracted data was made for neuroimaging modalities for which comparison between trauma subtypes was feasible, taking the studies' numbers and sample sizes into account.

**Results:** The systematic literature search yielded 25 publications. Sexual abuse was associated with structural deficits in the reward circuit and genitosensory cortex and amygdalar hyperreactivity during sad autobiographic memory recall. Emotional maltreatment correlated with abnormalities in fronto-limbic socioemotional networks. In neglected individuals, white matter integrity and connectivity were disturbed in several brain networks involved in a variety of functions. Other abnormalities, such as reduced frontal cortical volume, were common to all maltreatment types.

**Conclusions:** There is some evidence for long-term differential effects of trauma subtypes on the human brain. The observed alterations may result from both protective adaptation of and damage to the brain following exposure to threatening life events. Though promising, the current evidence is incomplete, with few brain regions and neuroimaging modalities having been investigated in all subtypes. The comparability of the available evidence is further limited by the heterogeneity of study populations regarding gender, age and comorbid psychopathology. Future neuroimaging studies should take this potentially differential role of childhood trauma subtypes into account.

## Introduction

### Rationale

In the past few decades research has amply demonstrated the detrimental effects that early-life stress, particularly childhood maltreatment, can have on the developing brain, with effects continuing into adulthood (1). Two recent meta-analyses report a reduction of hippocampal volume (2, 3). They also found a volume reduction of frontal cortical structures, as well as structural abnormalities in the amygdala, albeit the latter only in psychiatric populations. Besides these structural deficits, abnormal task-related activity has been reported in brain regions involved in emotion processing, such as the anterior cingulate cortex, amygdala and hippocampus, and in executive functions, such as the prefrontal cortex and basal ganglia (4). Communication between these brain regions might also be impaired due to structural deficits in the white matter tracts connecting them.

**Figure 1:**
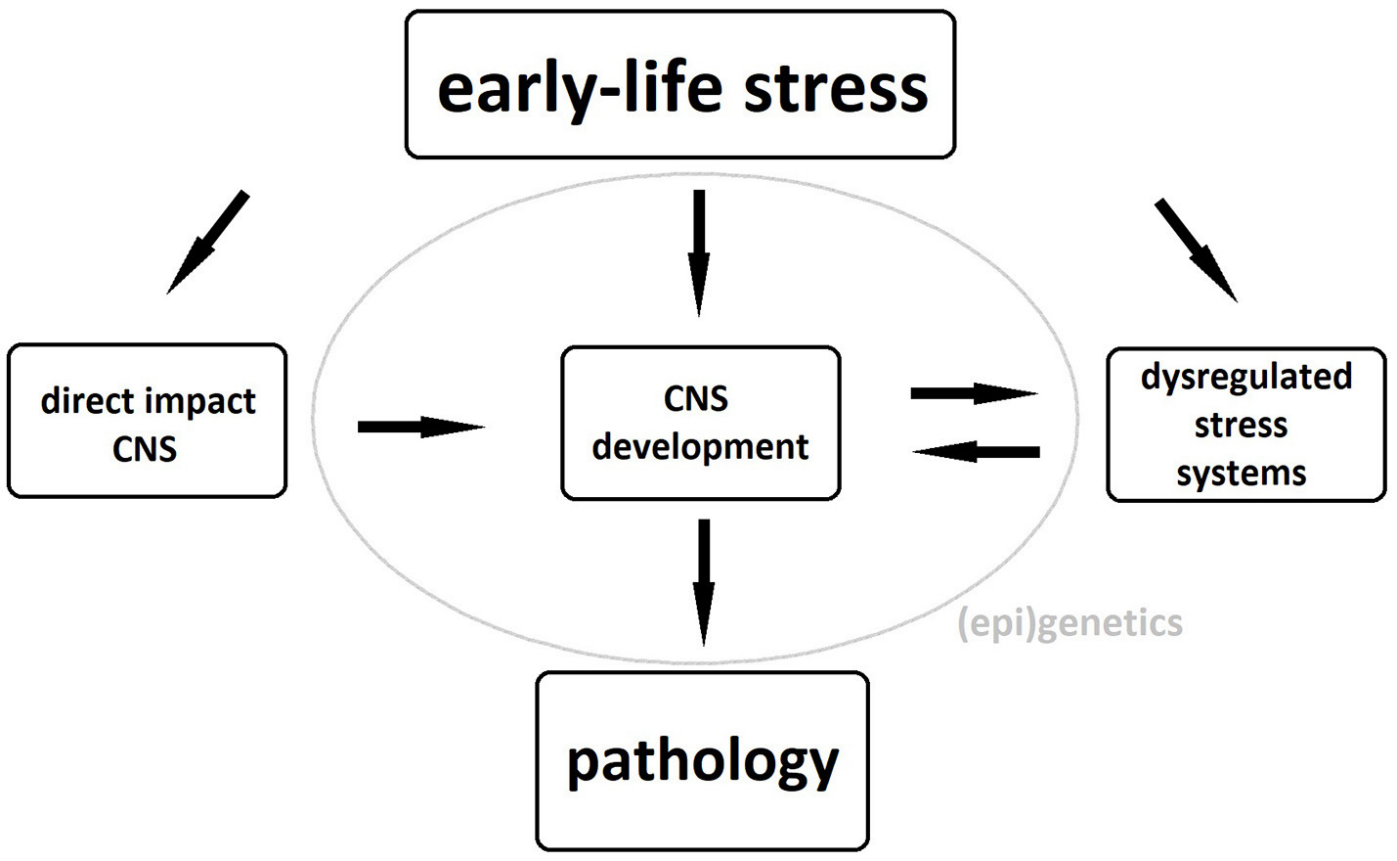
Biological pathways underlying the association between early-life stress and psychopathology. CNS, central nervous system.

The exact pathophysiological mechanism underlying these neuronal changes remains unclear, although dysregulation of stress systems, particularly of the hypothalamo-pituitary-adrenal (HPA) axis, plays an important role. Exposure of the developing brain to abnormal levels of glucocorticoids, the HPA axis' end products, can cause both structural and functional brain abnormalities and, if the latter occur in brain areas involved in the regulation of the HPA axis, these will further add to its dysregulation (5, 6). Gene-environment interactions as well as epigenetic phenomena such as glucocorticoid receptor methylation further strengthen the link between childhood maltreatment and HPA-axis dysregulation in adulthood (7, 8). These structural and functional brain alterations as well as the maladaptive stress response constitute an increased risk of psychopathology in maltreated individuals possibly due to a reduced ability to distinguish between threatening and safe cues during fear conditioning (9). A schematic representation of the biological pathways linking early-life stress to pathology can be seen in Figure 1.

Prolonged exposure to any type of stressor during childhood may thus result in brain injury, particularly in those regions that are sensitive to stress during brain development such as the hippocampus, amygdala, and prefrontal cortex (10). This *damage* inflicted to the brain might then lie at the base of neuroimaging findings of childhood trauma in general. However, evidence on a cognitive-behavioral level suggests the existence of differential effects of trauma subtypes (e.g., sexual abuse and emotional neglect) as well. In chronic fatigue syndrome, sexual abuse was the only childhood trauma subtype predictive of fatigue symptoms and physical functioning (11). Physical trauma types (i.e., physical abuse or neglect) and sexual abuse have been associated with reduced executive functioning, working memory, and intelligence scores in a psychiatric population (12) as well as reduced cognitive flexibility in healthy adults (13). These associations were not found for emotional abuse or neglect. Emotional maltreatment and physical abuse were predictive of later externalizing, aggressive behavior, whereas physical neglect was associated with internalizing, withdrawn behavior (14). On the other hand, emotional neglect was the only trauma subtype not associated with self-injury according to a recent meta-analysis (15). Subtypes also differentially affect the development of pathological personality traits, with emotional abuse being associated with cluster C, maternal neglect with cluster A and physical abuse with antisocial, narcissistic, and paranoid traits (16).

In addition, childhood emotional maltreatment is more associated with the later development of depressive symptoms than physical or sexual abuse (17–19). Interestingly, whereas adolescent unipolar depression appears to be associated with a history of maltreatment, particularly emotional, findings regarding bipolar depression are less consistent (20). Reported brain abnormalities in pediatric unipolar depressive disorder include a reduction of gray matter volume in the caudate nucleus and hippocampus, whereas bipolar disorder was associated with widespread frontolimbic and callosal white matter deficits (21), all of which are also associated with childhood maltreatment. Perhaps specific trauma subtypes lead to specific structural and functional brain alterations, which may then underlie the abovementioned differential cognitive-behavioral effects, and associated psychopathology, of childhood trauma subtypes.

Indeed, reductions of hippocampal and amygdalar volume reported in the previously mentioned meta-analysis by Paquola et al. (3) were largest in studies investigating the effects of physically and sexually abused victims, whereas lateral prefrontal cortical volume reductions were most apparent in studies investigating any type of trauma (abuse and/or neglect). Observing that victims of childhood abuse showed a reduction of amygdalar and hippocampal volumes, whereas neglected individuals had an increased amygdalar volume, Teicher and Samson (1) provided further important evidence for subtype-specific brain effects in their review. Also, they found some subtypes to be associated with abnormalities in sensory cortices involved in the processing of maltreatment-specific stimuli (e.g., lower thickness in the genital representation field of the somatosensory cortex in sexually abused individuals). This led to the hypothesis that brain alterations associated with childhood trauma might be adaptive, in line with the latent vulnerability model described by McCrory and Viding (22, 23). In this model, certain neurobiological alterations occur in response to maltreatment. Initially, these adaptations allow the young person to cope with the harmful rearing conditions. However, in the long run they may lead to maladaptive behavior in other (normative) environments, thus forming a latent vulnerability for the development of (psycho)pathology. For example the reduced thickness of the genital somatosensory cortex seen in victims of sexual abuse might modify the processing of adverse sensory input from the genital region (1). This can initially help the individual cope with the abuse, but in a later stage may lead to sexual dysfunction. Such an *adaptation* of the brain in relation to abuse-specific cues might better explain differential effects of trauma subtypes as opposed to a more general *damaging* of the brain by high levels of glucocorticoids in response to any stressor. However, as was already stressed by Teicher and Samson (1) the two hypotheses needn't necessarily be mutually exclusive. They point out that some brain alterations are associated with multiple trauma types and can thus be considered adaptive to more than one maltreatment context, e.g., changes in corticolimbic regions that are responsible for the processing of emotions. On the other hand, the adaptation to particular environmental demands associated with specific trauma subtypes might also be facilitated by neuroplastic (as opposed to neurodegenerative) mechanisms triggered by glucocorticoid receptor activation (24).

Although evidence of subtype-specific brain effects is accumulating, a systematic review of differential neuroimaging findings of the major trauma subtypes (i.e., sexual abuse, physical maltreatment, emotional maltreatment, and neglect) is as yet lacking. Previous meta-analyses and reviews were unable to provide clear evidence of such type-specific effects due to the paucity of evidence (2) and the failure of many clinical trials to correct for the frequent co-occurrence of multiple types of childhood maltreatment (1, 3).

### Objectives

In the present systematic review we focused exclusively on neuroimaging studies investigating long-term effects of specific, well-defined trauma types on the human brain whilst adjusting for the effects of other subtypes. We expected to find differential effects in distinctive brain regions that are associated with the processing of maltreatment-specific information (i.e., *adaptational* changes). These effects include somatosensory cortical deficits in physical and sexual abuse but also structural and functional abnormalities in regions involved in emotional processing (e.g., the anterior cingulate cortex and amygdala) in emotional maltreatment subtypes. We also expected increased functioning in brain regions involved in sensory processing in individuals with a history of childhood neglect due to a general absence of stimulation and an increased dependence on the limited bandwidth of information received in early life.

## Methods

The PRISMA guidelines (25) were adopted in the reporting of the methodology and results of this systematic review (Supplementary Table 8).

### Search strategy

A literature search was conducted in the PubMed and PsycINFO databases using the search terms (child abuse OR childhood abuse OR child sexual abuse OR childhood sexual abuse OR child neglect OR childhood neglect OR child maltreatment OR childhood maltreatment OR childhood adversit^*^ OR early life adversit^*^ OR “childhood trauma” OR “early life trauma”) AND (brain imaging OR magnetic resonance imaging) while applying a filter for human studies. In PubMed, terms between quotation marks yielded results for the literal term, whereas terms standing alone yielded wider search results (e.g., brain imaging yielded results for both “neuroimaging,” “Neuroimaging”[Mesh], and the combined terms “brain” AND “imaging,” thus including a broad range of neuroimaging modalities such as structural and functional MRI, diffusion tensor imaging, etc.). No limit was placed on the date or language of publication. The last search was conducted on 7 February 2018. This search yielded 1,134 PubMed and 463 PsycINFO papers. Also, a manual search was performed in the reference lists of selected papers and of relevant review articles retrieved by the database. This led to the further inclusion of one paper (26).

### Selection criteria

Animal studies, non-original and unpublished research papers were excluded as well as studies not reporting any type of neuroimaging outcome. Also excluded were studies that did not involve a direct comparison (either in primary or secondary analyses) of the outcome variables for exposed and unexposed individuals to either emotional, physical, or sexual childhood maltreatment while controlling for the effects of other subtypes either by excluding individuals with a history of these other types or by statistical correction. Papers investigating multiple subtypes were included provided they looked into the specific effects of each of these subtypes while controlling for the others. Childhood trauma subtypes were defined according to the criteria of the Fourth National Incidence Study (NIS-4; Table 1) (27) in which study maltreatment events are only considered childhood abuse if they cause actual harm or pose a serious risk of harm to a child up to 18 years of age and occur within the household setting (i.e., perpetrated by a family member or caregiver). One study on peer verbal abuse (28) was accordingly excluded (no household context).

**Table 1:** NIS-4 criteria for childhood trauma subtypes.

**Trauma subtype**	**Definition**
Sexual abuse	Any sexual act with a minor, including sexual penetration, molestation with genital contact, attempted sexual abuse with physical contact, child prostitution or pornography and exposure to sexually explicit material or voyeurism
Physical abuse	Hitting a child with hands or an object, kicking, punching, throwing, deliberately dropping, shaking, grabbing, dragging, pushing or pulling, or otherwise causing actual or threatened physical harm
Physical neglect	The refusal of custody or the deliberate failure to provide or seek needed care, supervision, nutrition, clothing, shelter, and personal hygiene or other disregard of a child's physical needs and safety
Emotional abuse	Verbal assaults or other abuse, threats, terrorization, administration of unprescribed substances or close confinement
Emotional neglect	Inadequate nurturing and affection, deliberate failure to provide or seek needed care for emotional-behavioral problems, allowing substance abuse or maladaptive behavior, overprotectiveness, inappropriately advanced expectations, inadequate structure and exposure to maladaptive behaviors and environments or domestic violence

We defined maltreatment as abuse, neglect or both of a particular type, where for instance emotional maltreatment can encompass both emotional abuse and neglect. Other types of early-life adversities such as loss of a parent, institutionalization, and poverty were not specifically addressed in our search because these dire circumstances do not fit our definition of maltreatment, while they also constitute a very heterogeneous group of life events that are difficult to assess in the light of the lack of objective measuring scales and the fact that they are not consistently controlled for in childhood trauma research. To include as many papers as possible, no a priori selection was made based on the language of the article, with none of the papers screened having to be excluded on account of a language barrier.

### Data extraction

Data was extracted from the selected papers by the first author. These included all neuroimaging outcomes compared across trauma subtype groups and comprised structural MRI, diffusion tensor imaging, and task and resting-state functional MRI findings. Both positive and null findings were taken into account. When a study did not report on effects in a priori defined regions of interest, this was considered a null finding. For whole-brain investigations, only the results reported in the original paper were considered. Information regarding study design, sample size, sample characteristics (mean age, gender distribution, psychiatric comorbidity), assessment method of traumatic childhood events (e.g., questionnaires, personal accounts, child protection service records) and applied statistical methods was also extracted from the various papers.

Given the nature of the research question and studies collected (cross-sectional neuroimaging findings after non-randomized exposure to childhood trauma), many of the risk of bias domains as assessed by the Cochrane Collaboration's tool (29) are not applicable to our data (e.g., selection bias, performance bias, and attrition bias). Differentiation between studies based on the risk of a reporting bias was difficult because pre-specified protocols were not available for any of the included studies. The risk of detection bias, on the other hand, was high in all but three studies reporting a blinding of the outcome assessors (30–32). Because these studies' results were not different from the results of other studies reporting on the same imaging modality in the same region of interest, this difference in risk of bias did not influence our synthesis and interpretation of the data.

## Results

### Study selection and characteristics

**Figure 2:**
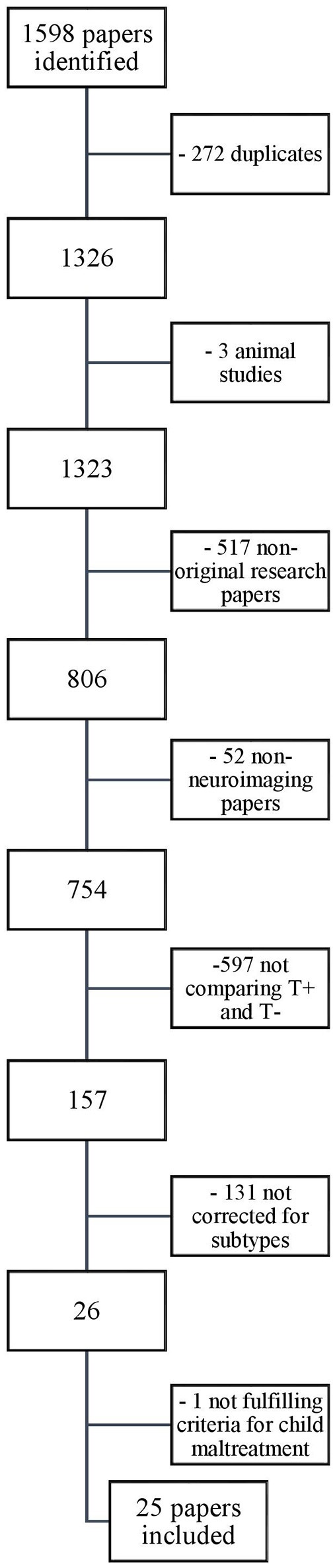
Selection of papers.

Our search yielded a total of 1,598 papers. These were screened for eligibility by the first author based on their titles and abstracts, and in case of doubt, on the methodological section in the full text of the article. Thousand five hundred and seventy-three papers were excluded based on the in- and exclusion criteria described above. The reasons for exclusion, with the relevant number of papers excluded, are provided in the flowchart depicted in Figure 2. The 25 remaining papers that were included in our review are listed in Table 2.

**Table 2:** Overview of included studies.

**References**	***n***	**Mean age (range^a^) in years**	**Investigated trauma types**	**Trauma assessment**	**Neuroimaging modality**
(31)	43	19.8 (18–22)	SA^2^^,^^3^	Interview (TAQ)	structural MRI
(30)	153	21.9 (18–25)	PA and EM^2^	CTQ, VAQ, TAI, CTS	Structural MRI
(33)	265	39.9 (18–70)	SA, PA, EA, EN^2^	ELSQ	Structural MRI
(34)	51	27.0 (18–45)	SA and EA	CTQ-SF	Structural MRI
(35)	60	- (18–45)	Neglect	CTQ-SF	Structural MRI
(32)	166	12.2 (5–19)	SA, PA, PN, and EM	Patient records	Structural MRI
(39)	37	19.7 (18–22)	SA^3^	TAI	Structural MRI
(42)	45	21.7 (17–26)	PA	Interview	Structural MRI
26	52	21.7 (19–24)	EM	TAI and VAQ	Structural MRI
(38)	145	36.9 (18–59)	EM^2^	NEMESIS	Structural MRI
(39)	42	21.9 (18–25)	PA	TAI	T2-weighted structural MRI
(40)	32	21.5 (18–25)	EA	VAQ and TAI	Diffusion tensor imaging
(41)	47	22.1 (18–25)	EN	TAI and CTQ	Diffusion tensor imaging
(42)	56	14.9 (11–17)	SA, PA, PN, EA, and EN	CTQ	Resting-state fMRI
(43)	22	29.8 (23–37)	SA, PA, PN, EA, and EN	CTQ-SF	Resting state fMRI
(44)	88	38.3 (18–60)	EM^2^	NEMESIS	Resting state fMRI
(45)	58	28.1 (11–46)	Neglect	CTQ-SF	Resting state fMRI
(46)	106	13.7 (12–15)	EN	CTQ	fMRI with reward processing task
(47)	31	16.1 (15–17)	EA^1^	VAQ	fMRI with gender identification of emotional faces task
(48)	20	35.8 (20–53)	PA	CTQ	fMRI with gender identification of emotional faces task
(49)	53	20.1 (19–21)	EM^2^	CAMEEI	fMRI with emotion regulation task
(50)	112	36.4 (18–59)	EM^2^	NEMESIS	fMRI with gender identification of emotional faces task
(51)	194	37.3 (18–58)	EM^2^	NEMESIS	fMRI during emotional word encoding and recognition
(52)	46	18.5 (15–23)	SA, PA, PN, and EM	CTQ-SF	fMRI with cyberball paradigm
(53)	24	40.7 (24–60)	SA, PA, EA, and neglect	CATS	fMRI with negative mood induction task

SA, sexual abuse; PA, physical abuse; PN, physical neglect; EA, emotional abuse; EN, emotional neglect; EM, emotional maltreatment; CTQ, Childhood Trauma Questionnaire; VAQ, Verbal Abuse Questionnaire; CAMEEI, Cambridge Early Experience Interview; TAI, Traumatic Antecedents Interview; CTS, Conflict Tactics Scales; TAQ, Traumatic Antecedents Questionnaire; ELSQ, Early Life Stress Questionnaire; CATS, Child Abuse and Trauma Scale; NEMESIS, NEMESIS trauma interview; CTQ-SF, Childhood Trauma Questionnaire—Short Form.

1 Not corrected for general neglect;

2 Not corrected for PN;

3 Not corrected for EA.

a *When age range was not explicitly stated in the original paper, ranges were derived from the 95% confidence interval obtained from the data*.

Of the included papers, only one reported on a longitudinal trial comparing neuroimaging findings across multiple time points (46). All other studies had cross-sectional designs. Sample sizes varied considerably, ranging from 20 to 265 participants. Four studies focused on neuroimaging effects of childhood trauma in adolescents (mean ages ranging from 12.2 to 16.1 years), with the other studies being conducted in adults with a history of childhood trauma. The majority described mixed gender samples, with two studies reporting on an exclusively male and four an exclusively female sample. Psychiatric comorbidity (mostly major depressive disorder (MDD) and anxiety disorders) was reported in 18 of the 25 studies, of which eight did so for the maltreated group only and not for the comparison group. Nine studies investigated a single maltreatment subtype (sexual abuse in two, physical abuse in three, emotional abuse in two, and emotional neglect in two studies), and another nine studies focused on higher-order subtype categories such as emotional maltreatment (i.e., emotional abuse and/or neglect) and general neglect (i.e., emotional and/or physical neglect) (seven and two studies, respectively). Seven studies looked into neuroimaging effects of multiple trauma subtypes, six of which compared subtypes directly through a between-groups ANOVA (*n* = 1) or multiple linear regression with all subtypes as predictors (*n* = 5). Most studies reported on physical abuse (*n* = 10), followed by sexual abuse and emotional maltreatment (*n* = 9) and emotional abuse (*n* = 7), while physical and emotional neglect subtypes were evaluated in four and neglect in general in three reports. Finally, the imaging modality in all studies was MRI, with 10 reporting structural, eight task-related fMRI, and eight resting-state or functional connectivity effects. One study used T2-weighted structural MRI to investigate regional cerebral blood volume in particular and two other studies diffusion tensor imaging to explore white matter tract integrity. Some studies reported multiple imaging modalities.

### Neuroimaging findings of trauma subtypes

To facilitate their comparison, neuroimaging findings (i.e., brain volume, activity, network connectivity, regional blood flow, and white matter integrity and density) are reported as either increased, decreased or unchanged as compared to the non-traumatized control state. A visual summary of all neuroimaging findings per trauma subtype is provided in the Supplementary Tables S1 (SA), S2 (PA), S3 (PN), S4 (EM), S5 (EA), S6 (EN), and S7 (general neglect).

**Figure 3:**
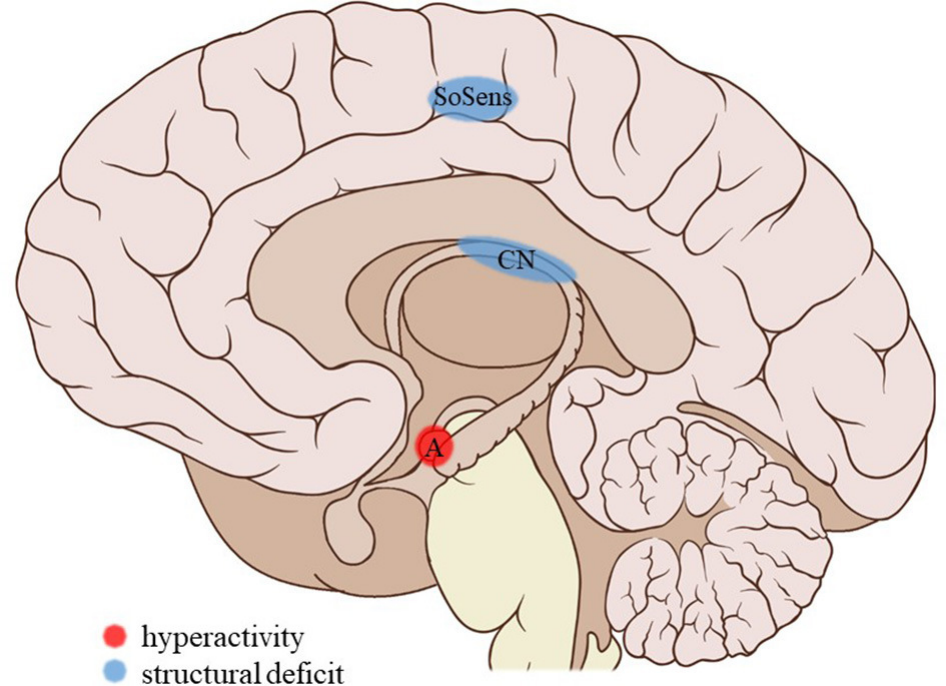
Neuroimaging findings associated with SA. SoSens, genital somatosensory cortex; CN, caudate nucleus; A, amygdala. Adapted from: Patrick J. Lynch, medical illustrator; C. Carl Jaffe, MD, cardiologist. https://creativecommons.org/licenses/by/2.5/

**Figure 4:**
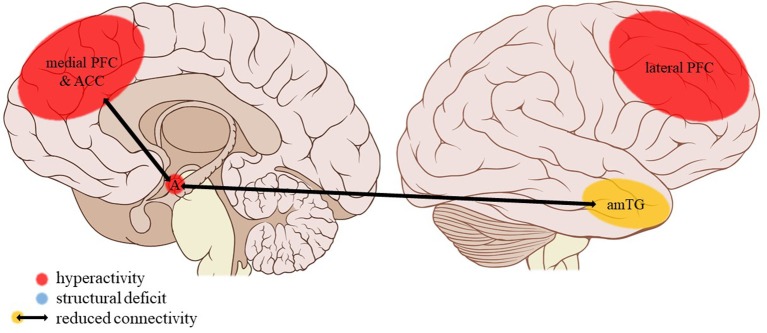
Neuroimaging findings associated with EM. PFC, prefrontal cortex; ACC, anterior cingulate cortex; A, amygdala; amTG, anterior middle temporal gyrus. Adapted from: Patrick J. Lynch, medical illustrator; C. Carl Jaffe, MD, cardiologist. https://creativecommons.org/licenses/by/2.5/

**Figure 5:**
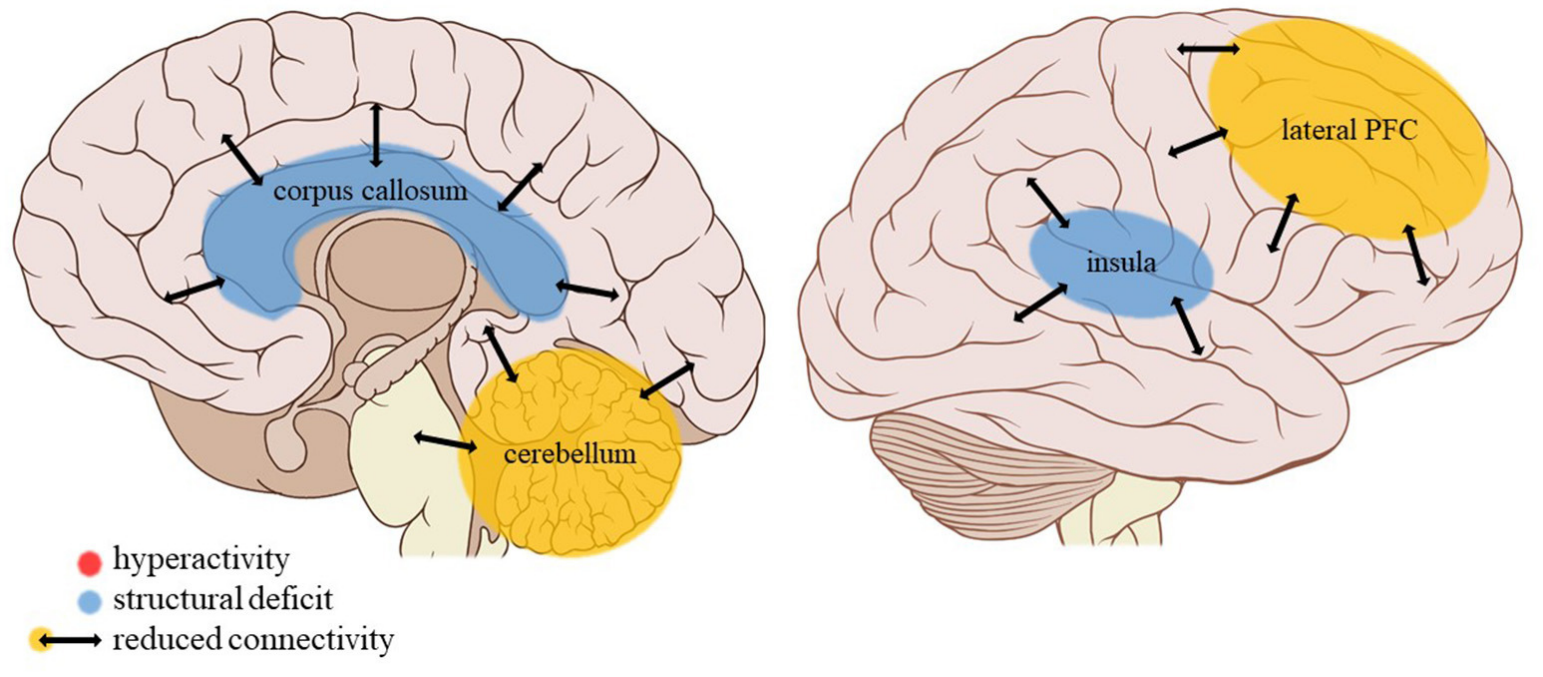
Neuroimaging findings associated with neglect. PFC, prefrontal cortex. Adapted from: Patrick J. Lynch, medical illustrator; C. Carl Jaffe, MD, cardiologist. https://creativecommons.org/licenses/by/2.5/

To allow actual comparisons of subtype-specific effects, a given neuroimaging outcome (e.g., amygdalar volume) should be reported for at least two different subtypes. This was only possible for structural MRI (volume) and task functional MRI (activity) and not for connectivity or white matter integrity outcomes. A summary of the most important findings is given in Table 3 and in Figures 3–**5**. We will next discuss the results per trauma subtype.

**Table 3:**
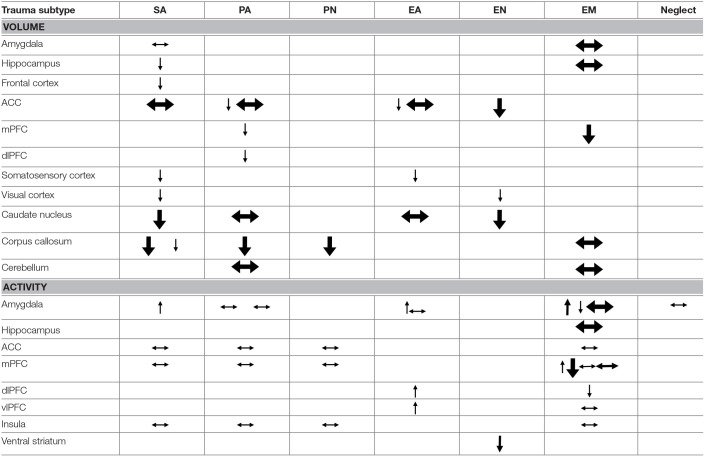
Structural and task-related functional comparison of trauma subtypes.

*Each arrow represents a significant effect reported in one of the included studies. Arrows pointing upwards or downwards represent an increase or decrease, respectively, in volume or activity of the associated brain structure. Horizontal arrows represent findings of unchanged volume or activity in maltreated individuals as compared to non-maltreated counterparts. The thickness of the arrows is determined by the size of the study sample on which the effect of the particular subtype was investigated (sometimes only a subset of the entire study sample). Cut-off points for sample sizes were n < 60 (small), 60 = < n < 120 (medium) and n > = 120 (large) [based on sample sizes of studies cited by Teicher and Samson (1, 54)]. Conflicting findings are indicated by differentially oriented arrows alongside each other within a cell. Blank cells represent effects that were not investigated in any of the included studies. SA, sexual abuse; PA, physical abuse; PN, physical neglect; EA, emotional abuse; EN, emotional neglect; EM, emotional maltreatment in general*.

#### Sexual abuse

##### Limbic regions

Childhood sexual abuse (SA) was associated with a number of structural and functional deficits in the limbic system. SA density (i.e., the number of years of abuse during a developmental stage divided by the number of years in that stage) during the pre-school and prepubertal stages (age 3–5 and 9–10 years, respectively) was related to a decreased hippocampal volume in adulthood (31). Similarly, SA was associated with lower cortical thickness in the parahippocampal gyrus (34). SA density could not be linked to structural amygdalar deficits (31) but was a predictor of right amygdalar hyperreactivity to sad mood induction (53). No effects were reported on resting-state functional connectivity of the amygdala with the medial prefrontal cortex (mPFC) (42) or the left anterior middle temporal gyrus (43).

##### Cortical structures

Frontal cortical gray matter volume (including the inferior, middle, and superior frontal gyri, orbitofrontal cortex and cingulate gyri) negatively correlated with SA density at ages 14–16 years (31). However, a study comparing multiple subtypes found no effect of SA on the anterior cingulate cortical (ACC) volume (33). Other studies reported lower cortical thickness in the left genital representation field of the somatosensory cortex (34) and bilateral visual cortex, more precisely in the left middle occipital and fusiform gyri and the right lingual gyrus, not in the occipital pole or cuneus (36). Also, during social exclusion, no effects of SA were found on cortical activity in the ACC, mPFC, or insula (52).

##### Other regions

Evaluating a large, healthy sample whilst correcting for other early-life adversities, Cohen et al. (33) found the caudate nucleus (CN) volume of individuals reporting SA to be reduced. Two studies reported reductions in the volume of the corpus callosum. Andersen et al. (31) found callosal volume to be negatively correlated to SA density at ages 9–10 years, whereas Teicher et al. (32) found a significant reduction in the volume of the anterior midbody of the corpus callosum, though not of the total callosal volume, in children with a history of SA.

#### Physical maltreatment

##### Physical abuse

*Limbic regions:* Physical abuse (PA) did not predict amygdalar activity during sad mood induction (53) nor was it associated with changes in amygdalar activity when processing negative emotional faces (48) or resting-state functional connectivity between the amygdala and the mPFC (42) or the left anterior middle temporal gyrus (43).

*Cortical structures:* Overall, PA was not a significant predictor of ACC volume (33) nor was it associated with changes of mPFC, ACC, or insular activity during social exclusion (52). Looking at harsh corporal punishment (HCP) while excluding other forms of PA, Tomoda et al. (37) observed a reduction of the right mPFC volume and also, after lowering the criteria for statistical significance, of the left dorsolateral PFC (dlPFC) and right dorsal ACC (dACC) volumes in the mistreated group. Although T2-weighted structural MRI could not identify an effect of HCP in the ACC, T2 relaxation time (RT) was increased in the dlPFC (39), suggesting a reduction of regional blood volume.

*Other regions:* PA was not significantly associated with volumetric changes in the CN (33) or cerebellar lingula (30). However, a reduced volume of the callosal isthmus was reported in children, although total volume of the corpus callosum was unaffected (32). Activity in the midbrain was found to be positively associated with the severity of PA in outpatients with borderline personality disorder during a gender identification of emotional faces task while controlling for other trauma subtypes (48). HCP was also associated with higher T2 RT and thus a lowered regional cerebral blood volume in the CN, putamen, thalamus, nucleus accumbens, and substantia nigra but not in the globus pallidus or cerebellar hemispheres (39).

##### Physical neglect

*Limbic regions:* Of all trauma subtypes, physical neglect (PN) was most strongly positively correlated to resting-state functional connectivity between the amygdala and the left anterior middle temporal gyrus (43). PN has no apparent effects on resting-state functional connectivity between the amygdala and the mPFC (42).

*Other regions:* No effects of PN were found on ACC, insula or mPFC activity in response to social exclusion (52). Total callosal volume was reduced in children with a history of PN referred for psychiatric evaluation (32).

#### Emotional maltreatment

A number of studies examined emotional maltreatment in general in that they did not differentiate between the effects of emotional abuse (e.g., being scolded at, being unjustly punished) and emotional neglect (e.g., not receiving adequate emotional support). We will first describe these general findings and then the findings for emotional abuse and neglect separately.

##### Emotional maltreatment in general

*Limbic regions:* Hippocampal and amygdalar volumes appear to be unaffected by a history of childhood emotional maltreatment (EM) (38), as is their activity during the encoding of (positive, negative, and neutral) words (51). When asked to downregulate their emotional response to aversive film footage, individuals reporting childhood adversities pertaining to EM (i.e., lack of affectionate warmth and communication, family discord and occasional physical violence between family members) showed decreased amygdalar activity (49). This was not the case when asked to downregulate their emotional reactivity to pleasant stimuli or to passively view aversive or pleasant stimuli (49). However, it must be noted that, according to the trauma measure used in this study, emotional abuse was absent in 87% of the maltreated participants and therefore emotional neglect is overrepresented in this particular sample. Conversely, amygdala activation was increased in individuals reporting EM during a gender identification of emotional faces task (50). Amygdalar activity was not affected during the encoding of negatively, positively, or neutrally valenced words (51). Functional connectivity during the downregulation of negative emotions was reduced between the right but not the left amygdala and the bilateral inferior parietal cortex (49). Reductions of negative and positive resting-state connectivity of the right, not the left, amygdala were also reported for the superior division of the occipital cortex, precuneus, and cuneus and a large cluster extending from the left of the orbitofrontal cortex and insula to subcortical structures including the hippocampus and putamen, respectively (55).

*Cortical structures:* EM was associated with a bilateral volume reduction of the dorsal mPFC (dmPFC) extending into the anterior mPFC (38). A hypoactivity of mPFC was noted in emotionally maltreated participants during word encoding and recognition (51), whereas another study demonstrated a positive correlation between EM severity and mPFC but not ACC activity during a social exclusion task (52). EM had no effect on mPFC activity during gender identification of emotional faces (50). Activity in the right medial frontal gyrus (corresponding to the dmPFC) while downregulating negative emotional reactivity was also unaffected (49). A reduced resting-state functional connectivity was reported between the left but not the right dACC and the right precuneus, right angular cortex (negative connectivity), and a bilateral frontal cluster encompassing the mPFC, paracingulate gyrus, and frontal pole (positive connectivity) (44). The same study found no evidence of changes in PCC resting-state connectivity. Functional connectivity of the mPFC with the hippocampus and amygdala during emotional word encoding and recognition (51) and resting-state connectivity of the left dmPFC (44) were unaffected by a history of EM.

Activity in the middle frontal gyrus (corresponding to the dlPFC) but not in the right inferior frontal gyrus (corresponding to the ventrolateral PFC (vlPFC)) was lower in an emotionally maltreated group (with overrepresentation of EN) during the downregulation of emotional reactivity to aversive but not to positive stimuli or when either positive or negative stimuli were being processed (49). The same study did not find an effect of EM on middle frontal gyrus (dlPFC) functional connectivity during these conditions.

When asked to downregulate emotional reactivity, emotionally maltreated participants showed hypoactivation of the posterior left middle temporal gyrus for aversive but not for pleasant stimuli and also not while they were exclusively processing either stimulus type (49). No effects of EM were recorded on insular activity during exposure to social exclusion (52) nor on left middle temporal gyrus connectivity during the downregulation of emotional reactivity to or merely processing positive or negative stimuli (49).

*Other regions:* Investigating the effects of EM (i.e., witnessing domestic violence and experiencing parental verbal abuse) in young adult women on the volume of the cerebellar lingula, Anderson et al. (30) found no effects. Also, and contrary to other trauma subtypes, there was no association between callosal volume and EM in children referred for psychiatric evaluation (32).

##### Emotional abuse

*Limbic regions:* Verbal abuse, a form of emotional abuse (EA), was associated with amygdalar hyperactivation during gender identification of faces with negative but not positive emotional expressions (47). In a negative mood induction task, however, no effects of EA were found on amygdalar activity (53). Verbal abuse severity scores correlated negatively with connectivity between the right amygdala and the rostral ACC and ventromedial PFC (vmPFC) when a gender identification task was performed on faces with negative emotional expressions (47). Likewise, EA was the only trauma subtype negatively correlated with resting-state connectivity between the amygdala and medial PFC (mPFC) (42). Resting-state connectivity between amygdala and the left anterior middle temporal gyrus was positively correlated with childhood trauma, which effect was driven by EA severity scores (after PA and EN) (43).

*Cortical structures:* EA was associated with lower cortical thickness in both the left (ventral) ACC and PCC in one study (34). However, another study comparing multiple types of childhood trauma and adversities (e.g., parental divorce, death of a parent or close family member, bullying) found no effect of EA on total ACC volume (33). Activity in the bilateral ventrolateral and right dorsolateral PFC was found to correlate positively with verbal abuse severity during gender identification of negative but not positive emotional faces (47). Lower cortical thickness of the face region of the somatosensory cortex and bilateral precuneus was associated with EA (34).

*Other regions:* EA was not significantly associated with CN volume (33). Fractional anisotropy was reduced in the left body of the fornix, the left arcuate fasciculus and the left fusiform gyrus in young adults reporting parental verbal abuse (40).

##### Emotional neglect

*Limbic regions:* Amygdalar resting-state connectivity with the mPFC was unaffected by emotional neglect (EN) (42). However, like PN and EA, EN was positively associated with amygdala and left anterior middle temporal gyrus baseline resting-state connectivity (43).

*Cortical structures:* A study comparing multiple trauma types reported smaller ACC volumes only in individuals reporting having witnessed domestic violence (WDV; a form of EN) in childhood (33). WDV was also associated with lower cortical thickness in a number of occipital regions, such as the right lingular gyrus (in the inferior occipital lobe), the bilateral secondary visual cortex and the left occipital pole (26), as well as with reduced fractional anisotropy in the left lateral occipital lobe, a region corresponding to the inferior longitudinal fasciculus (41). However, no WDV-associated changes were recorded in the primary or extrastriate visual cortex, the fusiform gyrus, the superior, middle and inferior occipital gyri, or the cuneus (26).

*Other regions:* A longitudinal study reported an association between EN and a blunted development of ventral striatal activity during reward processing (46). WDV was also found to be a significant predictor of smaller CN volume (33).

#### General neglect

##### Limbic regions

Amygdalar activity during negative mood induction did not appear affected by general neglect (experiencing a negative home environment with parental substance abuse, parental discord, emotional, and physical neglect) (53). Hippocampal white matter density, however, was increased in adults with MDD and a history of childhood neglect as compared to peers without such a history (35). Also comparing adult individuals with MDD and a history of moderate to severe childhood neglect to peers with MDD with no or less severe neglect, Wang et al. (45) observed a reduction in limbic connectivity strength (a measure of centrality in whole-brain functional networks) during resting-state encompassing the amygdala, hippocampus, and parahippocampal gyrus.

##### Cortical structures

Wang et al. (45) also observed a strong reduction in resting-state connectivity strength in the prefrontal cortex of their participants with MDD having suffered moderate to severe childhood neglect as compared to non-maltreated peers, particularly in the dlPFC and vlPFC and in the dorsomedial PFC (dmPFC) but not in the vmPFC. Insular connectivity strength was also reduced in this sample, whereas Peng et al. (35) found white matter density to be increased in sub-lobar, extra-nuclear regions, among which the insula, with white matter density in the bilateral inferior parietal cortices also being negatively correlated with CTQ neglect scores.

##### Other regions

The two studies comparing adults with MDD and a history of childhood neglect with non-maltreated peers with MDD also report an increased white matter density in the thalamus and CN (35) but a decreased resting-state functional connectivity strength of these structures in the maltreated group (45), while the functional connectivity strength in the cerebellum was also reduced (45).

## Discussion

### Summary of main findings

The results of our review confirm the existence of differential effects of childhood trauma subtypes on regional brain structures, activity and connectivity. The comparative findings for volume and activity are summarized in Table 3.

As becomes clear from Table 3, SA distinguishes itself from other subtypes by structural deficits within the reward circuit as well as amygdalar hyperreactivity (as found during recall of sad autobiographic memories). Effects specific to emotional maltreatment types include widespread abnormalities in fronto-limbic activity and connectivity. Neither PA nor neglect appears to have differential effects. However, the latter subtype does show widespread deficits of white matter integrity and connectivity in several large brain networks (not shown in Table 3). Other changes, such as reduced frontal cortical volume, are common to all maltreatment types. Still, it needs to be noted that Table 3 also illustrates that few findings are corroborated by more than one study and furthermore that neuroimaging outcomes were rarely investigated for all trauma types, as witnessed by the many blank cells. Hence, many uncertainties still exist and our results need to be interpreted with caution. We will next discuss the results per brain region and trauma subtype.

#### Limbic regions

##### Hippocampus

A reduction of hippocampal volume is one of the most consistently reported neuroimaging correlates of general childhood adverse experiences (1, 2), although a recent study with one of the largest samples (*n* = 3,036) ever used in similar investigations found no such effect (56). Our literature search yielded only two studies that looked into the effects of specific trauma subtypes, namely SA and EM. Whereas, SA was associated with a reduction of hippocampal cortical volume, EM was not. A recent meta-analysis demonstrated an effect of psychopathology on the association between hippocampal volume and childhood maltreatment that appeared to be mainly driven by studies investigating SA (2). Another meta-analysis of studies comparing abused and non-abused groups found the largest differences in hippocampal volume for SA/PA subtypes (3). SA was also associated with a reduced volume of the parahippocampal gyrus, which connects the hippocampus to the default mode network (57) and is, among other cognitive functions, involved in episodic memory (58). Deficits in short-term memory and recall were earlier reported in sexually abused individuals (59). Perhaps the abovementioned structural deficits in the memory network lie at the base of this early finding.

In conclusion, although hippocampal structural deficits are reported in victims of SA, the current evidence is too scarce to draw any firm conclusions as to the specificity of these effects.

##### Amygdala

Amygdalar volume was unaffected by SA and EM, which is in line with the results from a recent meta-analysis (2) that found no volume effects for any of the childhood trauma subtypes investigated.

PA does not appear to have an effect on amygdalar volume or activity. SA, on the other hand, is associated with amygdalar hyperreactivity to negative mood induction, which was not present in EA, PA, or general neglect. Rather than merely reflecting a high attributed salience to negative emotional stimuli in the populations studied (60), this finding might also be explained by the nature of the mood induction task used in which the participants were asked to re-experience sad personal life events by reading autobiographical scripts. Normally, the amygdala and hippocampus interact during emotional memory processing (61) and their activity and connectivity increase during emotional autobiographical recall (62). However, given the hippocampal structural deficits following SA, this balanced interplay might be disturbed. For example, amygdalar hyperreactivity is related to hippocampal hypoactivity in rats with memories consistent with posttraumatic stress disorder (PTSD) (61).

As we hypothesized, several studies report amygdalar activity and connectivity disturbances related to emotional maltreatment subtypes. However, these findings are complex. Firstly, emotional trauma types are associated with amygdalar hyperactivity during face processing. Emotional facial cues are known to activate the amygdala (63), especially in maltreated individuals (64). Arguably, emotional facial expressions have particular salience in the context of emotional trauma, given the important role of non-verbal communication in emotional human interaction. Amygdalar hyperreactivity to emotional faces in EM victims might then indicate an *adaptive* increased alertness for potential threat signaled by them. Indeed, altered amygdala activation to threat has been documented in traumatized individuals and might be a latent vulnerability for the development of psychopathology (22). The reduced amygdalar connectivity with the mPFC, rostral ACC, and insula, all of which are also involved in emotion regulation (65–68) and visual processing areas such as the occipital gyrus, cuneus, and precuneus (69–71) might add to amygdalar hyperreactivity due to reduced top-down control and visual information processing. Interestingly, parental verbal abuse (PVA), a form of EA, is also associated with reduced white matter integrity in the arcuate fasciculus and left fusiform gyrus, fiber bundles that are involved in language (72, 73) and face processing (74, 75). Thus, white matter structural deficits in EA are also situated in regions key to social interaction. EN and EA, like PN but not PA or SA, were associated with an increased connectivity to the anterior middle temporal gyrus, which plays a role in multimodal semantic processing (76) besides being activated in response to emotional faces (77). This increased connectivity might also facilitate processing of environmental threats within a social context (e.g., negative facial expressions). Interestingly, a study looking into the correlations of age at onset and duration of different trauma subtypes with amygdalar reactivity to emotional faces only found changes in activity for the EM and neglect subtypes, not for PA or SA (78). Although a definite conclusion on the specificity of these findings to EM or neglect cannot be drawn due to the current paucity of data and the prevalent co-occurrence of different trauma subtypes that is seldomly corrected for, the results of this study and our findings of amygdalar hyperreactivity to emotional faces predominantly within these subtypes are in line with each other.

Secondly, the experience of emotions as such did not cause amygdalar hyperreactivity in emotionally maltreated individuals. However, amygdalar recruitment during active downregulation of negative mood was less pronounced than it was in non-maltreated counterparts. Considering that, surprisingly, the participants reporting EM were better at downregulating their negative mood, they could be expected to also experience less distress and hence show less amygdalar activation (49). The reduction in functional connectivity between the amygdala and the inferior parietal cortex involved in evaluating emotional conflict (when displayed emotions do not correspond to the context) (79) might also increase the efficiency of emotion regulation during negative mood induction by reducing the conflicting informational stream.

Thirdly, when emotional words need to be classified according to their valence, amygdalar activity is apparently not affected by a history of EM. The authors (51) suggest that word classification is a cognitive process requiring activation of prefrontal brain areas, as opposed to the more emotionally-demanding face processing task associated with limbic activation.

Together, these findings indicate higher amygdalar reactivity in emotionally maltreated individuals in interpersonal contexts (i.e., while processing facial expressions) combined with a general reduction of connectivity with other structures guiding conscious emotional processing. Given that amygdalar activity was not increased in emotionally maltreated participants when they experienced negative mood or needed to classify emotional words, emotion regulation appears to be relatively intact in non-interpersonal emotional contexts.

General neglect is associated with a reduction of limbic connectivity strength, whereas emotional and physical neglect are each associated with an increase of anterior middle temporal gyrus and unchanged amygdala—mPFC resting-state connectivity. Perhaps the lack of emotional input during childhood minimizes the need for a strongly interconnected emotional regulation network but does place a higher demand on semantic processing in order for neglected individuals to make sense of the few environmental cues that are being recorded.

In summary, SA was associated with amygdalar hyperreactivity during the recall of sad autobiographic memories, a finding that was not reported for EA, PA, or general neglect. In EM, however, specific disturbances of the amygdalar social emotion network have been amply demonstrated (whereas non-social emotion processing appears to be intact). Neglected individuals also show disturbances of the amygdalar network but these are less specific.

#### Frontal cortex

Structural deficits of the frontal cortex, encompassing both the prefrontal and cingulate cortices, appear to be present in all trauma subtypes. This general finding might reflect the high sensitivity of the (pre)frontal cortex, which typically has a protracted development from childhood into early adulthood, to the detrimental effects of stress throughout childhood (4, 80). Frontal cortical volume reduction might therefore be the result of brain *damage* caused by HPA axis activation in response to (any type of) stress and as such may not be a particular feature of any specific trauma type. A previous meta-analysis indeed concluded that dlPFC volume reduction was most prevalently reported in studies investigating various trauma types and not one particular type (3).

Differential effects of trauma subtypes on frontal cortical structures also exist. We hypothesized that emotional maltreatment subtypes in particular would be associated with structural deficits in regions involved in the regulation of emotions, such as the ACC. One study comparing all types indeed found that only EN was associated with a reduction of ACC volume, but not EA, PA, or SA. The ACC is involved in the monitoring of emotional salience (pleasantness or aversiveness) of stimuli and the subsequent generation of appropriate response behavior (81). In the case of EN, a general lack of emotional input might render this monitoring function of the ACC less vital to the production of goal-directed behavior. Thus, a reduced volume of the ACC in victims of EN might be an *adaptation* of the brain in response to the low profit gained from continuously monitoring and appraising (absent) emotional cues (as opposed to, for example, EA environments, where adjusting behavior minutely to even the subtlest changes in emotional circumstances might prove to be an adaptational asset).

Frontal cortical activity and connectivity also seem to be differentially influenced by separate trauma subtypes. As we hypothesized, findings regarding the mPFC and ACC, which play an important role in the general appraisal and subconscious regulation of emotions (67), are most apparent in the EM subtype. Indeed, dmPFC hyperactivation to social exclusion is associated with EM but not SA, PA, or PN. Conversely, mPFC hypoactivation is seen in individuals reporting EM during the encoding of emotional words, independent of word valence. During emotion regulation, dmPFC activity remained unaffected. These conflicting findings seem to suggest that as a result of EM the medial frontal cortex may be particularly sensitive to emotional cues in a social context, contrary to a more general emotional mood regulation or the conscious classification of emotional words. Increased recruitment of the dmPFC during social exclusion might be a compensatory (*adaptive)* strengthening of top-down emotion regulation in victims of emotional maltreatment. In fact, these individuals had mood scores similar to those of non-maltreated controls after social exclusion, suggesting adequate emotion regulation. They were also better at mood regulation despite similar dmPFC activity and performed equally well in the word encoding paradigm despite medial frontal hypoactivity. Apart from being less specific to emotional processing in a social context, these processes are likely to be more conscious, deliberate forms of emotion processing that rather are related to lateral PFC (lPFC) functioning (67). Findings of reduced resting-state connectivity of the dACC with the mPFC as well as parietal structures including the angular cortex and precuneus involved in social cognition (82, 83) further strengthen the theory of altered emotional processing in a social context being specific to people having suffered EM as a child. Such deficits of the mPFC and ACC network connectivity are not present, for example, in physically or sexually abused individuals. Although *adaptive* to some extent, these functional alterations to the neurocircuitry of emotion processing in victims of emotional maltreatment may nevertheless form a latent vulnerability for the later development of psychopathology (22).

Participants reporting EM also show increased recruitment of the lPFC when processing negative but not positive emotional faces. Given the role of the lPFC in conscious emotional regulation (67) and taking into account the previously mentioned amygdalar hyperreactivity to negative emotional faces, these results suggest intensified top-down amygdalar control. Paradoxically, when instructed to downregulate negative emotional responses, dlPFC activity was lower in the EM group while they performed this task better than their non-maltreated peers. As mentioned previously, amygdalar activity was also reduced during this task, suggesting there is a lesser need for recruitment of the lPFC. The unchanged dlPFC functional connectivity seen in EM is indicative of a normal interaction with the amygdala during emotion processing. Neglect, on the other hand, is associated with a reduction of lPFC connectivity strength.

In summary, frontal cortical volume reduction appears to be a common feature for all trauma subtypes, while frontal top-down regulation of social emotional processing seems to be affected in individuals having suffered emotional maltreatment. Neglect is again associated with a more general reduction in frontal connectivity.

#### Sensory cortices

As we hypothesized, individuals reporting SA show a reduced volume of the genital representation field of the somatosensory cortex. EA, on the other hand, is associated with lower cortical thickness in the facial representation region. Reduced visual cortical volumes were also reported in both SA and WDV (a form of EN). WDV is also associated with reduced fractional anisotropy (FA) in the inferior longitudinal fasciculus, which connects the temporal and occipital lobe, adding further to the structural impairments of the visual cortex. This goes against our hypothesis of increased somatosensory processing in neglected individuals as an *adaptation* to the reduced sensory input.

However, these findings of lower sensory cortical thickness might still be an *adaptive* response of the brain to reduce the impact of abuse-related stimuli. Such defensive responses might specifically target brain regions involved in the processing of sensory information that is unique to a certain trauma subtype, as genital somatosensory stimuli are to SA. Other sensory cues might be more generally associated with several types of trauma. Visual stimuli would then, for example, have high salience both for SA victims (e.g., pornographic images) and for individuals reporting WDV (i.e., visually observing intraparental violence).

We did not identify studies reporting on structural or functional alterations in somatosensory brain regions in PA, so we were unable to confirm this hypothesis.

In conclusion, these findings suggest an impact of childhood trauma on cortices involved in processing trauma-related sensory information, whereby some sensory modalities are more specific to one particular subtype than to others.

#### Insula

Insular activity does not seem to be affected by any trauma type in social exclusion conditions. However, general neglect is related to a higher white matter density but reduced insular resting-state connectivity strength (a measure of centrality in the cerebral connectivity network). Apparently then, the efficiency of the information exchange through insular white matter bundles is decreased following childhood neglect. The insula monitors internal bodily sensations and is activated by emotionally salient information (67). Together with the ACC, it contributes to the initiation of adaptive behavior in response to these physical and emotional challenges, among other pathways by regulating the autonomic nervous system (81). However, neglected children experience a lack of physical as well as emotional input. Perhaps then the absence of these cues, and hence a lesser need for monitoring them and adjusting behavior accordingly, lies at the base of the lower connectivity strength of the insular network in neglected individuals. Interestingly, white matter informational exchange efficiency is similarly reduced in neglected individuals' thalamus, which normally conveys sensory information in the brain and plays a role in action monitoring and decision-making (84). Here the same mechanism of reduced necessity of sensory processing and appropriate behavioral adjustment in the absence of informational input might be at play.

Again, the finding of reduced insular network connectivity might be an *adaptive* alteration of the brain in order to reduce the experience of adverse bodily sensations (e.g., hunger, pain) or emotions (e.g., feelings of low self-worth, loneliness, rejection) that can accompany neglect, contrary to our hypothesized increased processing of these signals.

To summarize, neglect is associated with reduced connectivity in the insular network that is responsible for internal monitoring.

#### Reward circuit

The volume of the caudate nucleus (CN) is decreased in SA and EN, but not in PA or EA. The CN is part of the ventral striatum (VS), a very important and heavily interconnected component of the reward circuit (85). EN is also associated with blunted development of ventral striatal reactivity to positive versus negative reward stimuli in adolescents. General neglect is associated with reduced resting-state connectivity strength of the CN. Similar results were previously reported in the large neuroimaging study by Frodl et al. (56) who noted a reduction of CN volume in women for all trauma subtypes but particularly for EN and PN. These findings indicate that neglect, especially EN, is associated with both structural and connectivity deficits in the CN as well as deficiencies in (positive) reward processing. Given the involvement of the CN in cognitive reward processing, such as appropriate goal selection and outcome evaluation (86), the structural alterations observed in victims of childhood SA might contribute to the disturbed reward-based learning process previously reported for this trauma type (87).

In summary, SA and EN but not PA or EA appear to affect the volume of the CN possibly contributing to disturbed reward processing. Neglect is associated with more widespread disturbances of the reward circuit.

#### Cerebellum

Neglect is associated with a reduced resting-state connectivity strength in the cerebellum, a structure strongly connected to the somatomotor cortex as well as association networks including the default mode and executive control networks (88). Besides its well-known role in motor control, the cerebellum connects with error monitoring areas of the brain and contributes to working memory, learning and behavioral adaptation (89). Other trauma types such as PA or EM do not appear to have an effect on cerebellar volume (30) or blood volume (39). Given its role in error monitoring and behavioral adaptation, it is not surprising that neglect in particular is associated with reduced connectivity strength in the cerebellum due to the general lack of feedback (either positive or negative) characteristic of this trauma subtype.

Summarizing, childhood neglect is associated with a general reduction of connectivity strength in the cerebellum but due to the lack of studies on other trauma subtypes the evidence of its specificity is too limited to draw definitive conclusions.

#### Corpus callosum

The corpus callosum is an important white matter structure connecting the cerebral hemispheres and allowing communication between them (90). One study reports reductions in callosal volumes for SA, PA, and PN, but not for EM. PN is the only trauma type associated with total callosal volume reduction. Looking at SA and PA, volume reductions are exclusively seen in the anterior midbody and isthmus, respectively. These differential effects might also be interpreted as *adaptations* of the brain. Callosal thinning has been linked to behavioral deficits corresponding with cortical regions connected to the callosum (90). Therefore, information that is salient to a certain trauma subtype might be blocked by a volume reduction of specific callosal subregions connected to cortical areas processing this information. Specific cortical areas linking to the callosal anterior midbody and isthmus are the insula and overlying cingulate cortex, and the postcentral (somatosensory) and primary auditory cortex (90), respectively. Indeed, emotional processing and monitoring of the stress system play an important role in SA, vs. somatosensory and auditory cues in PA, whereas PN is associated with a more general reduced exchange possibly due to a more general lack of input.

Thus, in neglect there may be a general disturbance of interhemispheric communication, whereas in other subtypes this may be more limited to particular cortical sites such as the emotional processing areas in EA.

### Limitations

Although the evidence concerning differential neurobiological effects of childhood trauma types is promising, it is still limited. Firstly, few studies provide directly comparable neuroimaging outcomes across trauma subtypes, as is illustrated by the numerous blank cells in Table 3. The PA and EM subtypes were most frequently investigated, whereas PN, EN, and general neglect in particular were hardly studied. Also, the majority of studies merely looked at effects of one particular subtype (see Table 2), thus precluding conclusions regarding the specificity of the reported effects. However, six of the 25 studies we reviewed directly compared the effects of all trauma subtypes, with all clearly reporting differential effects. Findings were furthermore limited to a restricted number of regions of interest, more specifically, the amygdala and PFC, and imaging modalities, namely volumetric and task functional MRI. Thus, brain connectivity could not be compared across trauma types in regions other than the amygdala. Future research should therefore directly compare the effects of multiple trauma types using different imaging modalities in sufficiently large sample sizes to ensure adequate statistical power. Another limitation of the current evidence regarding long-term effects of both general and specific childhood trauma on the brain, is the scarcity of longitudinal studies exploring the causal relation between the occurrence of a traumatic event and alterations in the developing brain. Future study designs should include longitudinal data collection.

Next, the current review included studies reporting on either sexual, physical, or emotional maltreatment types (i.e., abuse and/or neglect) whilst correcting for the other types. However, some of the included studies only corrected for one type of emotional or physical trauma type (abuse or neglect) but not for the other (see Table 2). PN was the subtype most often not corrected for. The results should accordingly be interpreted with caution as they apply mostly to the larger maltreatment categories. Further investigation into the effects of each category (SA, PA, PN, EA, and EN) is needed.

Furthermore, across studies childhood trauma was measured in a variety of ways: through self-report questionnaires, (semi-)structured clinical interviews as well as patient health records. Also, definitions of subtypes were not always comprehensive and sometimes even differed slightly depending on the measuring scale used and/or the observer. These shortcomings limit the generalizability of the results. Future studies are therefore recommended to use explicit definitions, referring to official standards such as the NIS-4 (see Table 1), and preferentially use validated measuring scales with built-in subtype delineations such as the CTQ.

Lastly, this review did not take all known confounding variables into account. Previous studies suggested an effect of age of exposure and gender on neuroimaging findings in traumatized individuals (1). Also, the majority of the included studies reported on study samples with psychiatric comorbidity, such as MDD and PTSD, of which the associated neurobiological deficits have been amply documented (67). Another important consideration is the severity and frequency of maltreatment given its dose-response relationship with neuroimaging findings (91). The results of this review may then have been confounded because many of the studies we evaluated included samples with psychiatric comorbidity and unequal gender distribution while few looked into the moderating effects of age of exposure or severity of trauma. Furthermore, evidence of interactions of childhood stressful events with certain genotypes (and gender) is rapidly growing (92, 93). Therefore, future neuroimaging studies of childhood trauma should take these parameters into account and investigate their potential interacting effects on the brain as well.

## Conclusions

The current review provides preliminary evidence of differential effects of childhood trauma subtypes on brain structures, in terms of volume, activity, and connectivity during resting-state and during the execution of cognitive and emotional tasks. Sexual abuse was associated with structural deficits within the reward circuit and genital representation field of the somatosensory cortex as well as amygdalar hyperreactivity during the recall of sad autobiographic memories (see Figure 3). No apparent structural or functional effects were found for physical abuse. Effects of emotional maltreatment types include widespread abnormalities in fronto-limbic activity and connectivity, especially in networks involved in emotional processing in a social context (e.g., processing of facial expressions; see Figure 4). Finally, neglect was associated with widespread deficits of white matter integrity and connectivity in several large brain networks involved in a variety of functions (e.g., interoceptive monitoring; see Figure 5). Other deficits, such as reduced frontal cortical volume, are common to all maltreatment types. Subtype-specific trauma effects could result from protective *adaptation* (e.g., a reduction of the genital sensory cortical volume following sexual abuse), whereas more general trauma effects (e.g., a reduction of the frontal cortical volume) more likely stem from *damage* to the brain due to chronic exposure to threatening conditions and the accompanying glucocorticoid dysregulation. These mechanisms are not mutually exclusive, however (e.g., adaptive neuroplasticity induced by glucocorticoid receptor activation). Given the incompleteness of information on all trauma subtypes for many of the reported neuroimaging outcomes, notable uncertainties regarding the specificity of these brain alterations to particular trauma subtypes remain. Still, the current evidence is promising and urges differentiation between trauma types in further neuroimaging research. This will contribute to a better understanding of functional outcomes associated with specific trauma subtypes (e.g., higher risk for the development of major depressive disorder in emotionally maltreated individuals, sexual dysfunction in sexually abused individuals, and antisocial or narcissistic personality traits following physical abuse) and thus more personalized support and treatment for these individuals.

## Author contributions

All authors made substantial contributions to the study. LC and FV developed the concept and objectives of the review. LC, FV, LS, DV, and BP contributed to the design of the methodology. LC performed the acquisition of data under the supervision of FV. All authors contributed to the analysis and interpretation of the data, as well as the drafting and reviewing of the manuscript. All authors provide approval for publication of the content and agree to be accountable for all aspects of the work in ensuring that questions related to the accuracy or integrity of any part of the work are appropriately investigated and resolved.

### Conflict of interest statement

BP has received research funding (non-related to the work reported here) from Janssen Research and Boehringer Ingelheim. The remaining authors declare that the research was conducted in the absence of any commercial or financial relationships that could be construed as a potential conflict of interest.
