# Supplementary material for: Structural and Functional Brain Abnormalities Associated With Exposure to Different Childhood Trauma Subtypes: A Systematic Review of Neuroimaging Findings
Source: Front Psychiatry. 2018 Aug 3;9:329. doi: 10.3389/fpsyt.2018.00329 (PMC6086138; doi:10.3389/fpsyt.2018.00329)
Supplement: Supplementary file 1 [file Table_1.DOCX]

| Table S1: Neuroimaging findings in sexual abuse | | | | | | | | | |
| --- | --- | --- | --- | --- | --- | --- | --- | --- | --- |
|  | **Volume** | | | | | **Activity** | | **Resting state connectivity** | |
| **Brain region** | Andersen et al., 2008 | Cohen et al., 2006 | Heim et al., 2013 | Teicher et al., 2004 | Tomoda et al., 2009a | van Harmelen et al., 2014b^a^ | Yamamoto et al., 2017^b^ | Cisler et al., 2017 | Krause et al., 2016 |
| hippocampus |  |  |  |  |  |  |  |  |  |
| parahippocampal gyrus |  |  |  |  |  |  |  |  |  |
| amygdala |  |  |  |  |  |  |  | ^1^ | ^2^ |
| frontal cortex |  |  |  |  |  |  |  |  |  |
| ACC |  |  |  |  |  |  |  |  |  |
| mPFC |  |  |  |  |  |  |  |  |  |
| genital somatosensory cortex |  |  |  |  |  |  |  |  |  |
| visual cortex |  |  |  |  |  |  |  |  |  |
| insula |  |  |  |  |  |  |  |  |  |
| caudate nucleus |  |  |  |  |  |  |  |  |  |
| corpus callosum |  |  |  | ^3^ |  |  |  |  |  |
| ^a^social exclusion task  ^b^negative mood induction task  ^1^with mPFC  ^2^with anterior middle temporal gyrus  ^3^anterior midbody of corpus callosum | | | | | | | | | |
