# Supplementary material for: Structural and Functional Brain Abnormalities Associated With Exposure to Different Childhood Trauma Subtypes: A Systematic Review of Neuroimaging Findings
Source: Front Psychiatry. 2018 Aug 3;9:329. doi: 10.3389/fpsyt.2018.00329 (PMC6086138; doi:10.3389/fpsyt.2018.00329)
Supplement: Supplementary file 2 [file Table_2.DOCX]

| Table S2: Neuroimaging findings in physical abuse | | | | | | | | | | |
| --- | --- | --- | --- | --- | --- | --- | --- | --- | --- | --- |
|  | **Volume** | | | | **Activity** | | | **Resting state**  **connectivity** | | **Regional blood volume** |
| **Brain region** | Anderson et al., 2010 | Cohen et al., 2006 | Teicher et al., 2004 | Tomoda et al., 2009b | Nicol et al., 2015^a^ | van Harmelen et al., 2014b^b^ | Yamamoto et al., 2017^c^ | Cisler et al., 2017 | Krause et al., 2016 | Sheu et al., 2010 |
| amygdala |  |  |  |  |  |  |  | ^1^ | ^2^ |  |
| ACC |  |  |  |  |  |  |  |  |  |  |
| mPFC |  |  |  |  |  |  |  |  |  |  |
| dlPFC |  |  |  |  |  |  |  |  |  |  |
| insula |  |  |  |  |  |  |  |  |  |  |
| caudate nucleus |  |  |  |  |  |  |  |  |  |  |
| cerebellum |  |  |  |  |  |  |  |  |  |  |
| isthmus of corpus callosum |  |  |  |  |  |  |  |  |  |  |
| midbrain |  |  |  |  |  |  |  |  |  |  |
| putamen |  |  |  |  |  |  |  |  |  |  |
| thalamus |  |  |  |  |  |  |  |  |  |  |
| nucleus accumbens |  |  |  |  |  |  |  |  |  |  |
| substantia nigra |  |  |  |  |  |  |  |  |  |  |
| globus pallidus interna |  |  |  |  |  |  |  |  |  |  |
| ^a^fearful faces gender identification task  ^b^social inclusion task  ^c^negative mood induction task  ^1^with mPFC  ^2^with anterior middle temporal gyrus | | | | | | | | | | |
