# Supplementary material for: Structural and Functional Brain Abnormalities Associated With Exposure to Different Childhood Trauma Subtypes: A Systematic Review of Neuroimaging Findings
Source: Front Psychiatry. 2018 Aug 3;9:329. doi: 10.3389/fpsyt.2018.00329 (PMC6086138; doi:10.3389/fpsyt.2018.00329)
Supplement: Supplementary file 3 [file Table_3.DOCX]

| Table S3: Neuroimaging findings in physical neglect | | | | |
| --- | --- | --- | --- | --- |
|  | **Volume** | **Activity** | **Resting state connectivity** | |
| **Brain region** | Teicher et al., 2004 | van Harmelen et al., 2014b^a^ | Cisler et al., 2017 | Krause et al., 2016 |
| amygdala |  |  | ^1^ | ^2^ |
| ACC |  |  |  |  |
| mPFC | \|  \| \| --- \| |  |  |  |
| insula |  |  |  |  |
| corpus callosum |  |  |  |  |
| ^a^social exclusion task  ^1^with mPFC  ^2^with anterior middle temporal gyrus | | | | |
