# Supplementary material for: Structural and Functional Brain Abnormalities Associated With Exposure to Different Childhood Trauma Subtypes: A Systematic Review of Neuroimaging Findings
Source: Front Psychiatry. 2018 Aug 3;9:329. doi: 10.3389/fpsyt.2018.00329 (PMC6086138; doi:10.3389/fpsyt.2018.00329)
Supplement: Supplementary file 4 [file Table_4.DOCX]

| Table S4: Neuroimaging findings in emotional maltreatment | | | | | | | | | | |
| --- | --- | --- | --- | --- | --- | --- | --- | --- | --- | --- |
|  | **Volume** | | | **Activity** | | | | **Resting**  **state connectivity** | **Functional connectivity** | |
| **Brain region** | Anderson et al., 2010 | Teicher et al., 2004 | van Harmelen et al., 2010 | Schweizer et al., 2016^a^ | van Harmelen et al., 2013^b^ | van Harmelen et al., 2014a^c^ | van Harmelen et al., 2014b^d^ | van der Werff et al., 2013 | Schweizer et al., 2016^a^ | van Harmelen et al., 2014a^c^ |
| hippocampus |  |  |  |  |  |  |  |  |  |  |
| amygdala |  |  |  |  |  |  |  | ^1^ | ^3^ |  |
| mPFC |  |  |  |  |  |  |  |  |  | ^4^ |
| ACC |  |  |  |  |  |  |  | ^2^ |  |  |
| PCC |  |  |  |  |  |  |  |  |  |  |
| dlPFC |  |  |  |  |  |  |  |  |  |  |
| vlPFC |  |  |  |  |  |  |  |  |  |  |
| middle temporal gyrus |  |  |  |  |  |  |  |  |  |  |
| insula |  |  |  |  |  |  |  |  |  |  |
| lingula of cerebellum |  |  |  |  |  |  |  |  |  |  |
| corpus callosum |  |  |  |  |  |  |  |  |  |  |
| ^a^emotion regulation task  ^b^emotional faces gender identification task  ^c^emotional word encoding and recognition task  ^d^social exclusion task  ^1^negative connectivity with superior occipital gyrus, precuneus and cuneus, positive with orbitofrontal cortex, insula, hippocampus and putamen  ^2^negative connectivity with angular cortex and precuneus and positive with mPFC, paracingulate gyrus and frontal pole  ^3^with inferior parietal cortex  ^4^with hippocampus or amygdala | | | | | | | | | | |
