# Supplementary material for: Structural and Functional Brain Abnormalities Associated With Exposure to Different Childhood Trauma Subtypes: A Systematic Review of Neuroimaging Findings
Source: Front Psychiatry. 2018 Aug 3;9:329. doi: 10.3389/fpsyt.2018.00329 (PMC6086138; doi:10.3389/fpsyt.2018.00329)
Supplement: Supplementary file 5 [file Table_5.DOCX]

| Table S5: Neuroimaging findings in emotional abuse | | | | | | | | |
| --- | --- | --- | --- | --- | --- | --- | --- | --- |
|  | **Volume** | | **Activity** | | **Resting state connectivity** | | **Functional connectivity** | **White**  **matter**  **integrity** |
| **Brain region** | Cohen et al., 2006 | Heim et al., 2013 | Lee et al., 2015^a^ | Yamamoto et al., 2017^b^ | Cisler et al., 2017 | Krause et al., 2016 | Lee et al., 2015^a^ | Choi et al., 2009 |
| amygdala |  |  |  |  | ^1^ | ^2^ | ^3^ |  |
| ACC |  |  |  |  |  |  |  |  |
| PCC |  |  |  |  |  |  |  |  |
| vlPFC |  |  |  |  |  |  |  |  |
| dlPFC |  |  |  |  |  |  |  |  |
| somatosensory cortex |  |  |  |  |  |  |  |  |
| precuneus |  |  |  |  |  |  |  |  |
| caudate nucleus |  |  |  |  |  |  |  |  |
| fornix |  |  |  |  |  |  |  |  |
| arcuate fasiculus |  |  |  |  |  |  |  |  |
| fusiform gyrus |  |  |  |  |  |  |  |  |
| ^a^emotional faces gender identification task  ^b^negative mood induction task  ^1^with mPFC  ^2^with anterior middle temporal gyrus  ^3^with vmPFC and rostral ACC | | | | | | | | |
