# Supplementary material for: Structural and Functional Brain Abnormalities Associated With Exposure to Different Childhood Trauma Subtypes: A Systematic Review of Neuroimaging Findings
Source: Front Psychiatry. 2018 Aug 3;9:329. doi: 10.3389/fpsyt.2018.00329 (PMC6086138; doi:10.3389/fpsyt.2018.00329)
Supplement: Supplementary file 6 [file Table_6.DOCX]

| Table S6: Neuroimaging findings in emotional neglect | | | | |  |  |
| --- | --- | --- | --- | --- | --- | --- |
|  | **Volume** | | **Activity** | **Resting**  **state**  **connectivity** | | **White**  **matter**  **integrity** |
| **Brain region** | Cohen et al., 2006 | Tomoda et al., 2012 | Hanson et al., 2015^a^ | Cisler et al., 2017 | Krause et al., 2016 | Choi et al., 2012 |
| amygdala |  |  |  | ^1^ | ^2^ |  |
| ACC |  |  |  |  |  |  |
| occipital cortex |  |  |  |  |  | ^3^ |
| ventral striatum |  |  |  |  |  |  |
| caudate nucleus |  |  |  |  |  |  |
| ^a^reward processing task  ^1^with mPFC  ^2^with anterior middle temporal gyrus  ^3^inferior longitudinal fasciculus | | | | | | |
