# Supplementary material for: Structural and Functional Brain Abnormalities Associated With Exposure to Different Childhood Trauma Subtypes: A Systematic Review of Neuroimaging Findings
Source: Front Psychiatry. 2018 Aug 3;9:329. doi: 10.3389/fpsyt.2018.00329 (PMC6086138; doi:10.3389/fpsyt.2018.00329)
Supplement: Supplementary file 7 [file Table_7.DOCX]

| Table S7: Neuroimaging findings in neglect | | | |
| --- | --- | --- | --- |
|  | **Activity** | **Resting**  **state**  **connectivity** | **White**  **matter**  **density** |
| **Brain region** | Yamamoto et al., 2017^a^ | Wang et al., 2014 | Peng et al., 2013 |
| amygdala |  |  |  |
| hippocampus |  |  |  |
| PFC |  |  |  |
| insula |  |  |  |
| inferior parietal lobule |  |  |  |
| thalamus |  |  |  |
| caudate nucleus |  |  |  |
| cerebellum |  |  |  |
| ^a^negative mood induction task | | | |
